# Supplementary material for: Association Between Antecedent Statin Use and Decreased Mortality in Hospitalized Patients with COVID-19
Source: Res Sq. 2020 Aug 11:rs.3.rs-56210. Preprint. [Version 1] doi: 10.21203/rs.3.rs-56210/v1 (PMC7430584; doi:10.21203/rs.3.rs-56210/v1)
Supplement: Supplement [file supplementalfile.docx]

**SUPPLEMENTAL APPENDIX**

**Association Between Antecedent Statin Use and Decreased Mortality in**

**Hospitalized Patients with COVID-19**

**TABLE OF CONTENTS**

Page 2 Supplemental Table 1. ICD-10-CM codes used to classify baseline conditions

Page 3 Supplemental Table 2. Missing rate of laboratory markers

Page 4 Supplemental Figure 1. Distribution of the estimated propensity score for antecedent statin use, among patients who did and did not actually receive the treatment

Page 5 Supplemental Figure 2. Standardized mean differences in the unmatched and matched sample

Page 6 Supplemental Figure 3. Multivariable regression to examine association of antecedent statin use with primary endpoint (in-hospital mortality within 30 days) in study sample restricted to patients with history of hypertension, coronary artery disease and stroke or transient ischemic attack.

Page 7 Supplemental Figure 4. Multivariable regression to examine association of antecedent statin use (modified definition)* with primary endpoint (in-hospital mortality within 30 days)

**Supplemental Table 1. ICD-10-CM codes used to classify baseline conditions**

| **Comorbidity** | **ICD-10-CM codes** |
| --- | --- |
| Hypertension | I10-13, I15-16, O10.1-10.4, O10.9 |
| Diabetes | E08 -11, E13, O24.4 |
| Coronary Artery Disease | I21-25, Z98.61, Z95.1 |
| Heart Failure | I09.81, I11.0, I13.0, I50, I42 |
| Chronic Lung Disease | J40-47, J60, J66, J67.2, J67.8-67.9, J68.4, J84, G47.3 |
| Stroke/transient ischemic attack | I60-64, I69, H34.1, G45 |
| Atrial arrhythmias | I47.1, I47.9, I48.0-48.4, I48.91, i48.92, I49.9 |
| Chronic Kidney Disease | N03, N07-08, N11, N14, N18-19, N29, I12, I13, Z99.2, E10.22, E11.22, E13.22, E08.22, O10.3, D63.1 |
| Chronic Liver Disease | K70 – K77 |

**Supplemental Table 2. Missing laboratory markers in the propensity-matched cohort**

| **Laboratory marker** | **Statin Users (648)** | **Non-statin Users (648)** |
| --- | --- | --- |
| White blood cell count | 1 (0.1%) | 4 (0.6%) |
| Platelet count | 6 (0.9%) | 8 (1.2%) |
| Creatinine | 0 | 0 |
| AST | 7 (1.0%) | 10 (1.5%) |
| ALT | 11 (1.7%) | 12 (1.9%) |
| Hs-Troponin | 34 (5.2%) | 47 (7.3%) |
| Albumin | 5 (0.8%) | 11 (1.7%) |
| D-dimer | 92 (14.2%) | 111 (17.1%) |
| Ferritin | 38 (5.9%) | 59 (9.1%) |
| ESR | 80 (12.3%) | 74 (11.4%) |
| CRP | 38 (5.9%) | 43 (6.6%) |

ALT = alanine aminotransferase; AST = aspartate aminotransferase; CRP = C-reactive protein; ESR = erythrocyte sedimentation rate; hs-Troponin = high sensitivity Troponin T; IQR = interquartile range

|  |  |  |
| --- | --- | --- |

**Supplemental Figure 1. Distribution of the estimated propensity score for antecedent statin use, among patients who did and did not actually receive the treatment**


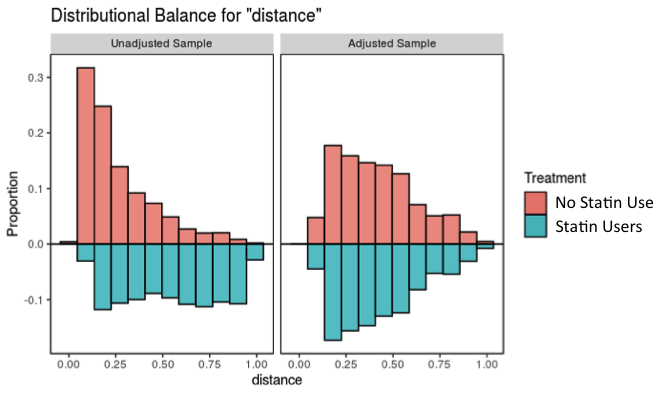


**Supplemental Figure 2**. **Standardized mean differences in the unmatched and matched sample**


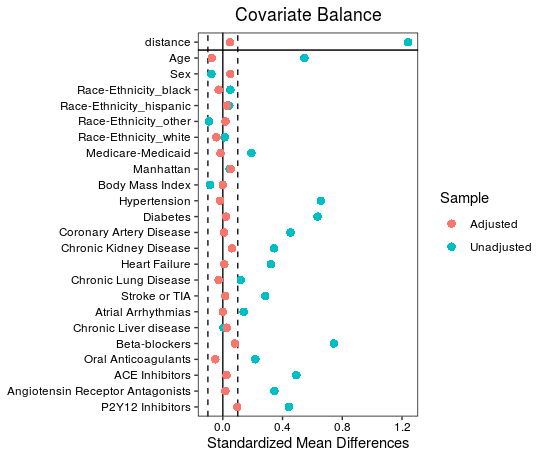


**Supplemental Figure 3. Multivariable regression to examine association of antecedent statin use with primary endpoint (in-hospital mortality within 30 days) in study sample restricted to patients with history of hypertension, coronary artery disease and stroke or transient ischemic attack.**


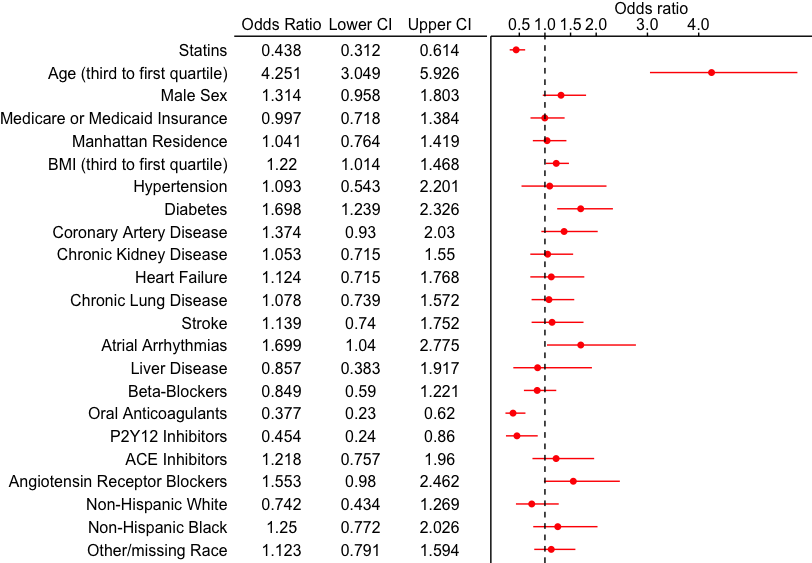


**Supplemental Figure 4. Multivariable regression to examine association of statin use (modified definition)* with primary endpoint (in-hospital mortality within 30 days)**

**
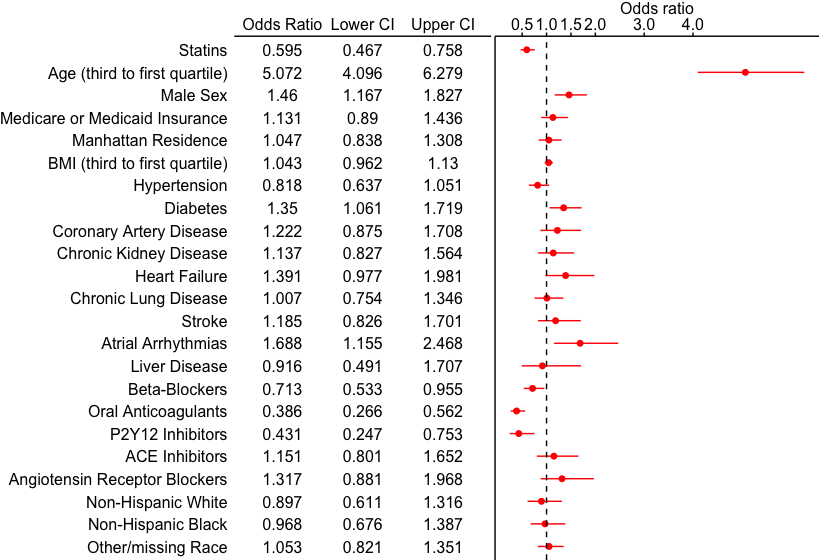
**

*In this analysis, patients were classified as statin users if they were either prescribed outpatient statin use per the electronic medical record or received statins during hospitalization
